# Aktualisierte ESAIC-Leitlinie zum postoperativen Delir beim Erwachsenen

**DOI:** 10.1007/s00101-024-01404-6

**Published:** 2024-04-22

**Authors:** Martin Söhle, Mark Coburn

**Affiliations:** Klinik für Anästhesiologie und Operative Intensivmedizin, Universitätsklinikum Bonn, Venusberg-Campus 1, Gebäude 22, 53127 Bonn, Deutschland

**Keywords:** Präoperatives Screening, Risikofaktoren, Dexmedetomidin, Burst Suppression, EEG-Monitoring, Preoperative screening, Risk factors, Dexmedetomidine, Burst suppression, EEG monitoring

## Abstract

Die aktualisierte Leitlinie der ESAIC zum postoperativen Delir (POD) umfasst insgesamt 13 Empfehlungen, darunter fünf mit dem Empfehlungsgrad „stark“: 1.) Die Erfassung der präoperativen POD-Risikofaktoren, 2.) die Optimierung des präoperativen Zustands, 3.) die Besprechung von Präventionsstrategien, 4.) die Durchführung einer nicht-pharmakologischen Multikomponenten-Intervention bei POD-Risikopatienten und 5.) die Nutzen-Risiko-Abwägung bei der prophylaktischen Gabe von Dexmedetomidin. Letzteres gilt insbesondere auf Grund der teils widersprüchlichen Datenlage und unterschiedlicher Einsatzgebiete (herzchirurgische versus nicht-herzchirurgische Patienten). Weiterhin wird die indexbasierte EEG-Überwachung der Narkosetiefe empfohlen, wobei auch weitere Parameter, wie die Burst Suppression und das Density Spectral Array mit einbezogen werden sollten. Wenn nicht-pharmakologische Maßnahmen versagen, sollte das POD mit Haloperidol therapiert werden. Hingegen wird der Einsatz von Benzodiazepinen nicht empfohlen.

## Abstract (en)

The updated ESAIC guideline on postoperative delirium (POD) comprises a total of 13 recommendations, including five with the recommendation grade “strong”: 1.) The assessment of preoperative POD risk factors, 2.) the optimisation of the preoperative condition, 3.) the discussion of prevention strategies, 4) the implementation of a non-pharmacological multicomponent intervention in patients at risk of POD and 5.) the risk-benefit assessment of the prophylactic administration of dexmedetomidine. The latter applies in particular due to the partly contradictory data situation and different areas of application (cardiac surgery versus non-cardiac surgery patients). Index-based EEG monitoring of the depth of anaesthesia is also recommended, whereby other parameters such as burst suppression and density spectral array should also be included. If non-pharmacological measures fail, POD should be treated with haloperidol. In contrast, the use of benzodiazepines is not recommended.

Im Jahr 2017 hat die European Society of Anaesthesiology and Intensive Care (ESAIC) erstmals eine evidenz- und konsensbasierte Leitlinie zum postoperativen Delir (POD) beim Erwachsenen herausgegeben [1]. Nun hat das Autorenteam um César Aldecoa und Claudia Spies in der Februarausgabe des *European Journal of Anaesthesiology* ein Update der Leitlinie publiziert [3]. Hierzu wurden 1768 Publikationen, die im Zeitraum vom April 2015 bis Februar 2022 veröffentlicht wurden, gesichtet und bewertet. Die aktualisierte Leitlinie umfasst 13 Empfehlungen, auf die im Folgenden kurz eingegangen wird. Trotz der Vielzahl an erschienenen Publikationen schwankt der Evidenzgrad der Empfehlungen zwischen „sehr niedrig“ und „moderat“, was die Notwendigkeit unterstreicht, die Forschungstätigkeit fortzusetzen und zu intensivieren, um die Erkenntnisse auf eine sichere Wissensbasis zu stellen.

Bei der präoperativen Anästhesieberatung wird empfohlen, bei älteren Erwachsenen ein Screening auf Risikofaktoren für POD durchzuführen (***Empfehlung 4.1****, niedrige Evidenz, starke Empfehlung*), wobei die folgenden 4 Risikofaktoren bewertet werden sollten: (1) höheres Alter (> 60 Jahre), (2) ASA-Score > 2, (3) Charlson Comorbidity Index > 2, (4) Mini-Mental State Examination (MMSE) Score < 25 Punkte (***Empfehlung 2.1****, moderate Evidenz, starke Empfehlung*). Hingegen wird die Verwendung von Biomarkern zur POD-Risikobeurteilung nicht empfohlen (***Empfehlung 3.4****, niedrige Evidenz, schwache Empfehlung*). Die Ergebnisse des Screenings auf POD-Risikofaktoren sollten im Behandlungsteam ausgetauscht sowie die Präventionsstrategien besprochen und in die Krankenakte eingetragen werden (***Empfehlung 4.2****, niedrige Evidenz, starke Empfehlung*). Weiterhin sollte auf die Bedürfnisse der Patienten eingegangen werden, um ihren präoperativen Status zu optimieren (***Empfehlung 4.1****, niedrige Evidenz, starke Empfehlung*).

Für den eigentlichen operativen Eingriff wird weder eine medikamentöse Prophylaxe eines POD empfohlen (***Empfehlung 3.1****, niedrige Evidenz, schwache Empfehlung*) noch ein bestimmtes Anästhesie- oder Operationsverfahren, um die Häufigkeit eines POD zu verringern (***Empfehlung 3.3****, niedrige Evidenz, schwache Empfehlung*). Falls man sich doch für den intra- oder postoperativen Einsatz von Dexmedetomidin **zur Prophylaxe** eines POD entscheidet, so sollten die zu erwartenden Vorteile gegen die wichtigsten Nebenwirkungen (Bradykardie und arterielle Hypotonie) abgewogen werden (***Empfehlung 3.2****, moderate Evidenz, starke Empfehlung*). Auf den ersten Blick scheinen sich diese beiden Empfehlungen (keine medikamentöse POD-Prophylaxe vs. Dexmedetomidin als POD-Prophylaxe) zu widersprechen, bei genauem Nachlesen werden die Hintergründe aber erläutert: Die gegenwärtige Evidenzlage lässt vermuten, dass es eine Patientengruppe, die von einer intra- und/oder postoperativen Dexmedetomidingabe profitieren könnte, gibt. Die Datenlage ist jedoch widersprüchlich, und somit ist bis dato unklar, welche Patientengruppe profitieren könnte, wobei es Hinweise gibt, dass dies v. a. ältere Patienten sein könnten. Weiterhin ist unklar, zu welchem Zeitpunkt und in welcher Dosierung Dexmedetomidin verabreicht werden sollte. Aufgrund des Nebenwirkungsprofils von Dexmedetomidin (Bradykardie und arterielle Hypotonie) und des Grundsatzes, dass eine Therapie zuerst keinen Schaden anrichten soll („first do no harm“), wurde entschieden, Dexmedetomidin für die POD-Prophylaxe nicht generell zu empfehlen. Die weiteren Details sind der aktualisierten Empfehlung und insbesondere ihrem Supplement zu entnehmen.

Bei allen Patienten, bei denen ein Delirrisiko besteht, sollte eine nichtpharmakologische Multikomponentenintervention durchgeführt werden (***Empfehlung 4.3****, moderate Evidenz, starke Empfehlung*). Diese zielt u. a. auf die Beseitigung von modifizierbaren POD-Risikofaktoren wie Anämie, Mangelernährung oder Dehydratation ab, wovon v. a. ältere, gebrechliche Patienten mit einer niedrigen funktionellen Reserve profitieren könnten. Diese Multikomponentenintervention umfasst beispielsweise Maßnahmen wie Stressabbau, Schlafförderung (z. B. Geräuschminderung/Ohrstöpsel, Augenbinden), reorientierende Maßnahmen (z. B. Bereitstellung von Brillen und Hörgeräten), frühzeitige Entfernung von Kathetern, frühzeitiger Kostaufbau und Mobilisierung. Hierbei ist die Effektivität der Einzelmaßnahmen nur schwierig zu beurteilen, jedoch scheint klar zu sein, dass ein Bündel aus Interventionen effektiver ist als einzelne Maßnahmen.

Eine indexbasierte EEG-Überwachung der Narkosetiefe wird weiterhin empfohlen, um das Risiko eines POD zu verringern (***Empfehlung 5.1****, niedrige Evidenz, schwache Empfehlung*). Es wird jedoch empfohlen, bei der Narkoseführung nicht nur den EEG-Index (Bispektraler Index, Narcotrend Index, Patient State Index, Entropie etc.) zu berücksichtigen, sondern weitere EEG Parameter wie Burst Suppression und Density Spectral Array miteinzubeziehen (***Empfehlung 5.2****, niedrige Evidenz, schwache Empfehlung*). Daher wird vorgeschlagen, Anästhesisten zu schulen, damit sie lernen, wie man Roh-EEG und Density Spectral Array interpretiert. Beispielsweise kann man in Ersterem ein Burst-Suppression-EEG-Muster und in Letzterem eine unzureichende Aktivität im α‑EEG-Band erkennen; beides Phänomene, die mit einem erhöhten POD-Risiko einhergehen und daher vermieden werden sollten.

Für die **Behandlung eines Delirs** gibt die Leitlinie Empfehlungen für den Einsatz insbesondere von Haloperidol, Benzodiazepinen und Dexmedetomidin:

Haloperidol sollte niedrig dosiert, kurzfristig und symptomorientiert verabreicht werden, wenn nichtpharmakologische Maßnahmen versagen (***Empfehlung 6.1****, sehr niedrige Evidenz, schwache Empfehlung*). Die Anwendung sollte in Bolusform und mit der niedrigstmöglichen Dosis (0,125–0,25 mg) erfolgen, wobei eine maximale Tagesdosis von 3 mg nicht überschritten werden sollte. Vorsicht ist bei Menschen mit vorbestehenden neurologischen Erkrankungen wie der Parkinson-Krankheit oder Lewy-Körperchen-Demenz geboten. Bei diesen sollten Antipsychotika nur mit Vorsicht oder gar nicht eingesetzt werden.

Die Evidenz für den Nutzen einer Benzodiazepintherapie zur Behandlung von POD-Symptomen ist sehr gering bis nicht vorhanden. Daher wird der Einsatz von Benzodiazepinen zur Behandlung des POD explizit **nicht** empfohlen (***Empfehlung 6.2****, sehr niedrige Evidenz, schwache Empfehlung*). Vom POD ist das Delir im Rahmen eines Alkoholentzugs abzugrenzen, bei dem Benzodiazepine symptomorientiert als Medikament der ersten Wahl empfohlen werden (in einer bolustitrierten Dosis, so niedrig wie möglich).

Auf den **prophylaktischen** Einsatz von Dexmedetomidin wurde bereits weiter oben eingegangen. Zum **therapeutischen** Gebrauch von Dexmedetomidin beim Auftreten eines POD konnte insbesondere bei nichtherzchirurgischen Patienten aufgrund unzureichender Evidenz keine Empfehlung ausgesprochen werden. Hingegen gibt es bei herzchirurgischen Patienten Hinweise, dass eine Therapie mit Dexmedetomidin die Dauer von POD und den Aufenthalt auf Intensivstation/im Krankenhaus sowie die Mortalität reduzieren könnte. Daher wird der Einsatz von Dexmedetomidin zumindest für herzchirurgische Patienten empfohlen (***Empfehlung 6.3****, sehr schwache Evidenz, schwache Empfehlung*). In seinem Rote-Hand-Brief vom 15.06.2022 weist das Bundesinstitut für Arzneimittel und Medizinprodukte auf das Risiko einer erhöhten Mortalität nach Gabe von Dexmedetomidin bei Intensivpatienten ≤ 65 Jahre hin [2] und bezieht sich hierbei auf die SPICE-III-Studie [4]. Diese umfasste ein breites Spektrum aus operativen und nichtoperativen Intensivpatienten und beschrieb bei der Altersgruppe der ≤ 65-Jährigen eine gesteigerte Mortalität v. a. bei **nicht**operativen Patienten.

Zusammengefasst enthält die aktualisierte Leitlinie insgesamt fünf starke Empfehlungen: Diese beinhalten erstens die Erfassung der präoperativen POD-Risikofaktoren (höheres Alter (> 60 Jahre), ASA > 2, CCI > 2, MMSE < 25), zweitens die Optimierung des präoperativen Zustands, drittens die Besprechung von Präventionsstrategien mit Eintrag in die Krankenakte, viertens die Durchführung einer nichtpharmakologischen Multikomponentenintervention bei POD-Risikopatienten und fünftens die Abwägung von Nutzen und Nebenwirkungen bei einer prophylaktischen Gabe von Dexmedetomidin.
